# Primitive neuroectodermal tumor of genital tract in hysterectomized patient: A case report

**DOI:** 10.4274/tjod.88714

**Published:** 2018-09-03

**Authors:** Betül Yakıştıran, Salih Taşkın, Cevriye Cansız Ersöz, Fırat Ortaç

**Affiliations:** 1 Ankara University Faculty of Medicine, Department of Obstetrics and Gynecology, Ankara, Turkey; 2 Ankara University Faculty of Medicine, Department of Pathology, Ankara, Turkey

**Keywords:** Ovary tumor, primitive neuroectodermal tumor, Ewing’s tumor

## Abstract

Primitive neuroectodermal tumors are high-grade malignant neoplasms. These are uncommon entities for the female genital tract. The treatment, management and follow-up period of Ewing’s tumors are not well-defined because of their rarity in the genital tract. Surgical debulking is the mainstay treatment in all cases. After debulking surgery, patients receive chemotherapy and/or radiotherapy and there is a relation between disease stage and survival. Herein, we present a case of ovarian primitive neuroectodermal tumor with a review of previously reported cases.

## Introduction

Primitive neuroectodermal tumors (PNETs), which are known as Ewing’s sarcoma (ES), are high-grade malignant neoplasms that develop from a group of neuroectodermal small round cells^(1)^. The typical locations of PNETs are around the skeletal system, but they can arise from any soft tissue^(2)^. ES is an uncommon condition in the female genital tract;^(3)^ ovarian tumors with primitive neuroectodermal components for postmenopausal women are extremely rare and only a few cases have been reported. Herein, we present a case of ovarian PNET with review of previously reported cases.

## Case Report

**Figure 1:**
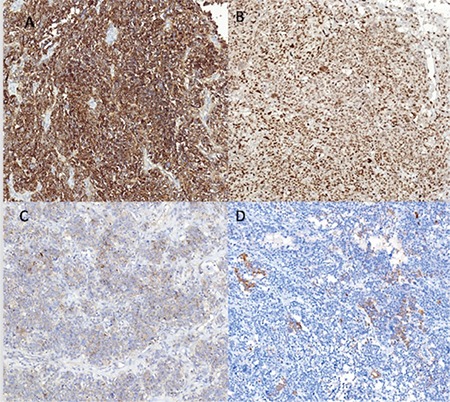
A) Strong membranous and cytoplasmic CD56 positivity (CD56*10), B) Nuclear Fli1 positivity (Fli1*10), C) Focal cytoplasmic SYNP staining (SYNP^*^10), D) Focal HMW+LMW CK positivity^*^10

**Figure 2:**
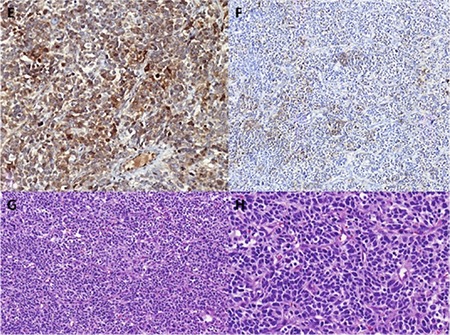
E) Neuron spesific enolase staining (^*^10), F) Epithelial membrane antigen staining (^*^10), G, H) Hematoxyline-eosin staining (^*^10)

A 64-year-old woman, gravida 2, para 2, presented with pelvic pain, which she had had for approximately four months. She underwent a ventro-suspension 25 years ago for uterine prolapse. However, a re-operation for uterine prolapse consisting of laparoscopy-assisted vaginal hysterectomy was performed 3 years ago. During this procedure, the uterus was separated from bilateral cornual regions and adnexae were left. The result of a pathologic evaluation was reported as benign for the uterus corpus material but wide cervical intraepithelial grade 3 neoplasia signs for the cervix were reported. A physical examination revealed a pelvic mass fixed to the left anterolateral abdominal wall. Abdominal magnetic resonance imaging revealed a huge mass in the pelvic cavity backward the bladder with irregular borders. The tumor markers were carbohydrate antigen (CA)-125; 269.7 kU/L (reference value; 0-35 kU/L). She underwent a debulking operation with bilateral salpingoopherectomy and total omentectomy, bilateral pelvic and paraaortic lymph node dissection, appendectomy, and aspiration for cytologic evaluation. The left ovarian mass had invaded the abdominal wall and resection of the fascia and part of the rectus abdominis muscle was needed; a polypropylene mesh was used to close the abdominal wall. There was no visible tumor after surgery. The tumor was characterized by a proliferation of small, round, primitive cells with a diffuse growth pattern. The cells had scant cytoplasm, irregularly-shaped and hyper-chromatic nuclei with coarse chromatin and a brisk mitotic rate. In some areas there were perivascular pseudorosette-like structures. The histology showed round cells with hyper chromatic nuclei and pleomorphisms, eosinophilic cytoplasm, very frequent mitosis, apoptosis, and focal necrosis. The tumor showed diffuse, strong, cytoplasmic and membranous CD56, nuclear Fli-1 positivity. Multifocal staining for neuron specific enolase (NSE) and mesothelin and focal high molecular weight (HMW)+low molecular weight cytokeratin (CK), epithelial membrane antigen (EMA), synaptophysin (SYNP), WT1 positivity was detected (Figure 1, 2). The tumor cells were also positive for p53. CD99, chromogranin A, CD45, inhibin, calretinin, CA-125, ER, PR, CK7, CK20, Moc31, Tag72, myogenin, S100, and destine were negative. The surgical specimens of one ovary, appendix, and omentum were interpreted as Ewing sarcoma/PNET after immunohistologic and histologic studies. The patient was referred to the medical oncology department and chemotherapy consisting of vincristine, cyclophosphamide, and cisplatin was started. Radiotherapy was not applied. The CA-125 value was 84.4 U/mL before her first chemotherapy. The patient completed six chemotherapies after surgery. There was no evidence of disease after 7 months of follow-up.

## Discussion

PNETs are uncommon entities especially for the female genital tract and the ovaries are the most common location^(1,2,3,4,5,6,7,8,9,10,11,12,13,14,15)^. It seems that the exact age for non-skeletal ES is not clear, but cases in the literature were seen between the second and third decades of the life. The first postmenopausal patient with ovarian PNET was reported by Fischer et al.^(16)^. According to our knowledge, this case is the second ovarian Ewing’s tumor to be diagnosed in the postmenopausal period. For our patient, invasion to the anterolateral abdominal wall may have been due to suspension to this region of the round ligament and adnexa during the operation for uterine prolapse many years ago. To date, nearly 30 ovarian ES cases have been reported and some of these are summarized in Table 1.^(6,7,8,9,10,11,17,18,19,20,21,22)^ PNETs have poor prognosis and due to the fact that tumors have an aggressive potential, survival periods are short. Two reported cases in literature had six years’ survival. Age at diagnosis is meaningful for five-year overall survival ranges. As reported in the case of Fischer et al.^(16)^ the patient was alive for six months. Therefore, our patient is one of the rare cases to be diagnosed in postmenopausal period and has the longest survival period. The treatment, management, and follow-up periods for Ewing’s tumors in the genital tract are not well-defined because of their rarity. Until recently, 18 cases of uterus corpus, 5 cases of cervix uteri, 3 cases of vulvar, and 4 cases of vaginal location have been reported^(23)^. Uterine abnormal bleeding and enlargement of the uterus size were the main symptoms reported for uterine corpus and cervix uteri ES-PNETs, and painless, nodular vulvar masses were typical for the vulvar or vaginal tumors. Among these cases, tumor markers had increased values in nearly all patients. Surgical debulking was the mainstay of treatment in all cases. After debulking surgery, patients received chemotherapy and/or radiotherapy. Although different chemotherapy agents were used for each patient in literature, generally therapies were designed as platinum-based^(2,6,10,11,15,23)^. Additionally ifosfamide, bleomycin, vincristine and doxorubicin, alternatively dacarbazine,^(6)^ and adriamycin^(5)^ were administered in some cases. The effect of radiotherapy has not been proved so there is need for more studies of cases of primary ovarian ES treatment. In the pathophysiologic pathway, the immature precursors of neural and glial cells from the embryonic period may proliferate and implant on the peritoneum and behave as malignant cells. However, other germ cells may persist and continue forming neural tube-like rosettes and medullary structures. All these stem from precursors in the neuroectoderm, and they are all  called neuroectodermal tumors of the ovary. The translocation between chromosomes 11 and 22-t(11;22) (q24;q12)-is the same genetic problem for the PNETs group^(8,9,10)^. The differential diagnoses of PNETs of the ovary include several primary and metastatic ovarian neoplasms such as juvenile granulosa cell tumors, lymphoblastic lymphoma (LBL), extrauterine endometrial stroll sarcoma, and serous ovarian carcinomas. The distinction between ES of the ovary and other tumors is made through immunohistochemistry studies. As seen in Table 2,^(12,13,14,15,24)^ on immunohistochemistry, diffuse membranous positivity for MIC2 (CD99), CD56 (neural cell adhesion molecule), HMW CK and FL1 led to the consideration of PNETs. Negativity for epithelial markers such as CK, EMA, desmin, and WT-1 led to the consideration of desmoplastic small round cell tumors (SRCTs). Positive staining of CD10, actin, and vimentin is considered as extrauterine stromal sarcoma, and negative staining is for PNET. Also, granulosa cell tumors are frequently reactive for CK and inhibit, although PNETs are non-reactive^(25)^. In immunohistochemistry, these tumors usually exhibit positivity for CD99, vimentin, and FLI-1. However, expression of many other markers can be found including NSE, SYNP, chromogranin, CD56, CD57, S-100, and neurofilament protein. In addition, some tumors have focal positivity for CK. CD99 is a sensitive marker for PNETs, but also positive for some other SRCTs such as lymphomas, rhabdomyosarcomas. Accordingly, these findings limit the specificity of this antibody. As in our case, CD99 may be negative in 10% of tumors^(26)^. The differential diagnosis is broad and includes neoplasms composed of “small blue round” cells, which can be encountered in the ovary; small cell carcinoma of hypercalcemic type; extrauterine endometrial stromal sarcoma; rhabdomyosarcoma; melanoma; desmoplastic round cell tumor; and lymphoma/leukemia. Small cell carcinoma of hypercalcemic type affects adolescents and young adults, typically between the ages of 9 and 43 years and is associated with hypercalcemia^(27)^. Extrauterine endometrial stromal sarcomas are typically positive for vimentin and smooth muscle actin, and most tumors stain for CD10. Our tumor was negative for CD10. Embryonal rhabdomyosarcomas may be CD99 and FLI-1-positive^(28)^. It is characterized by alternating hyper and hypocellular myxoid areas and shows small cells admixed with spindle cells that may contain cross striations. There were no areas like those described above in our tumor and also myogenin was negative in our tumor^(29)^. Melanoma may be composed of small cells but it often arises in association with ovarian cystic teratomas. It may show melamine pigment and more conventional areas. The characteristic histologic appearance of desmoplastic round cell tumors is peripheral palisading of basaloid cells, forming irregular islands that may show central necrosis, surrounded by a desmoplastic stroma. Expression of keratins and destine may be helpful in the differential diagnosis. Expression of FLI-1 by LBL might potentially lead to a misdiagnosis of LBL as ES/PNET because we found diffuse FLI-1 positivity; but even when growing in a diffuse pattern, lymphomas still may show admixture of lymphoid and myeloid cells in different stages of maturation^(28)^. Also CD45 was negative in our case. There was a relation between disease stage and survival as shown in Table 1. However, one reported case in which the patient was young and had advanced stage at diagnosis had 6 years’ disease-free survival after debulking surgery and adjuvant chemotherapy. Our patient had advanced stage disease and 7 months’ disease-free survival after completing the therapy. In conclusion, preoperative findings and survival results of ovarian ES may be similar to epithelial ovarian cancer. However, treatment of these tumors is not standardized due to their rarity.

## Figures and Tables

**Table 1:**
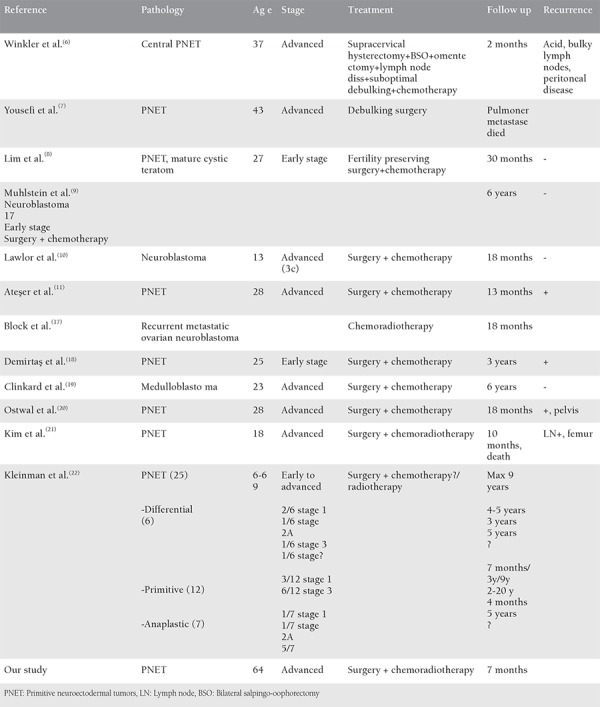
Clinicopathological features of primary ovarian Ewing’s sarcoma-primitive neuroectodermal tumor

**Table 2:**
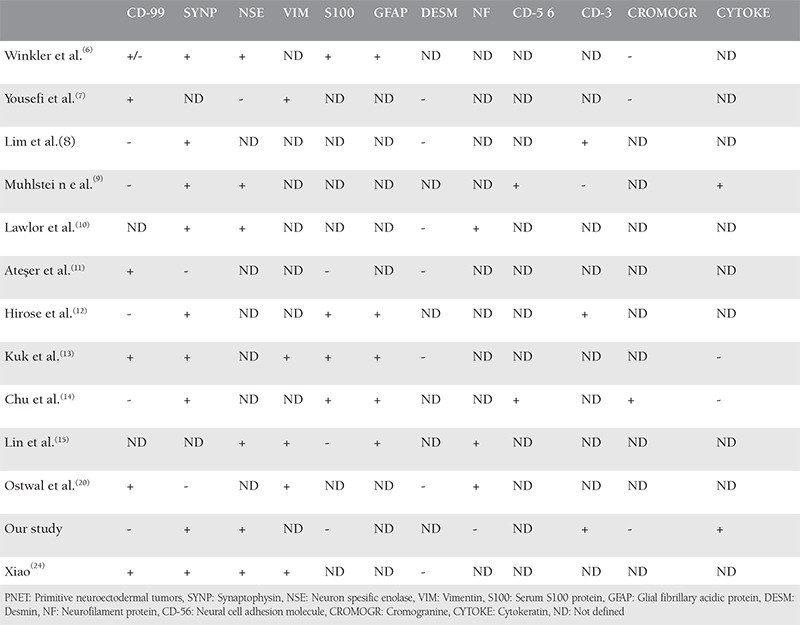
Immunohystochemical staining for ovarian primitive neuroectodermal tumors

## References

[1] Dizon AM, Kilgore LC, Grindstaff A, Winkler M, Kimball KJ (2013). High grade primitive neuroectodermal tumor of the uterus: A case report. Gynecol Oncol Case Rep.

[2] Niwa Y, Yamamuro O, Kato N, Tsuzuki T (2013). Two cases of primary ovarian neuroblastoma arising from mature cystic teratomas. Gynecol Oncol Case Rep.

[3] Blattner JM, Gable P, Quigley MM, McHale MT (2007). Primitive neuroectodermal tumor of the uterus. Gynecol Oncol.

[4] Snijders-Keilholz A, Ewing P, Seynaeve C, Burger CW (2005). Primitive neuroectodermal tumor of the cervix uteri: a case report-changing concepts in therapy. Gynecol Oncol.

[5] Boldorini R, Riboni F, Cristina S, Allegrini S, Valentini S, Muscarà M, et al (2010). Primary vulvar Ewing’s sarcoma/primitive neuroectodermal tumor in a post-menopausal woman: a case report. Pathol Res Pract.

[6] Winkler SS, Malpica A, Soliman PT (2015). Novel treatment of a central type, primitive neuroectodermal tumor of the ovary with postoperative pediatric medulloblastoma chemotherapy regimen: A case report and review of the literature. Gynecol Oncol Rep.

[7] Yousefi Z, Sharifhi N, Hasanzadeh M, Mottaghi M, Bolandy S (2014). Peripheral primitive neuroectodermal tumor of the pelvis. Iran J Med Sci.

[8] Lim YK, Ku CW, Teo GC, Lim SL, Tee CS (2013). Central primary primitive neuroectodermal tumor (cPNET) arising from an ovarian mature cystic teratoma in pregnancy: A case report and review of medical literature. Gynecol Oncol Case Rep.

[9] Muhlstein J, Rodriguez-Dahloff S, Marie B, Fouyssac F (2010). Primary ovarian neuroblastoma. J Pediatr Adolesc Gynecol.

[10] Lawlor ER, Murphy JI, Sorensen PH, Fryer CJ (1997). Metastatic primitive neuroectodermal tumor of the ovary: successful treatment with mega-dose chemotherapy followed by peripheral blood progenitor cell rescue. Med Pediatr Oncol.

[11] Ateser G, Yildiz O, Leblebici C, Mandel NM, Unal F, Turna H, et al (2007). Metastatic primitive neuroectodermal tumor of the ovary in pregnancy. Int J Gynecol Cancer.

[12] Hirose T, Nobusawa S, Kusanishi H (2015). Ovarian primitive-type neuroectodermal tumor composed of desmoplastic/nodular medulloblastoma-like and atypical teratoid/rhabdoid tumor components. Histopathology.

[13] Kuk JY, Yoon SY, Kim MJ, Lee JW, Kim BG, Bae DS (2012). A case of primitive neuroectodermal tumor of the ovary. Korean J Obstet Gynecol.

[14] Chu LH, Chang WC, Kuo KT, Sheu BC (2014). Primary primitive neuroectodermal tumor of the ovary. Taiwan J Obstet Gynecol.

[15] Lin CH, Lin YC, Yu MH, Su HY (2014). Primary pure large cell neuroectodermal carcinoma of the ovary. Taiwan J Obstet Gynecol.

[16] Fischer G, Odunsi K, Lele S, Mhawech P (2006). Ovarian primary primitive neuroectodermal tumor coexisting with endometrioid adenocarcinoma: a case report. Int J Gynecol Pathol.

[17] Block M, Gilbert E, Davis C (1984). Metastatic neuroblastoma arising in an ovarian teratoma with long-term survival. Case report and review of the literature. Cancer.

[18] Demirtaş E, Güven S, Güven ES, Baykal C, Ayhan A (2004). Two successful spontenous pregnancies in a patient with a primary primitive neuroectodermal tumor of the ovary. Fertil Steril.

[19] Clinkard DJ, Khalifa M, Osborned RJ, Bouffet E (2011). Successful management of medulloblastoma arising in an immature ovarian teratoma in pregnancy. Gynecol Oncol.

[20] Ostwal V, Rekhi B, Noronha V, Basak R, Desai SB, Maheshwari A, et al (2013). Primitive neuroectodermal tumor of ovary in a young lady, confirmed with molecular and cytogenetic results--a rare case report with a diagnostic and therapeutic challenge. Pathol Oncol Res.

[21] Kim KJ, Jang BW, Lee SK, Kim Bk, Nam SL (2004). A case of peripheral primitive neuroectodermal tumor of the ovary. Int J Gynecol Cancer.

[22] Kleinman GM, Young RH, Scully RE (1993). Primary neuroectodermal tumors of the ovary. A report of 25 cases. Am J Surg Pathol.

[23] Park JY, Lee S, Kang HJ, Kim Hs, Park SY (2007). Primary Ewing’s sarcoma-primitive neuroectodermal tumor of the uterus: a case report and literature review. Gynecol Oncol.

[24] Xiao C, Zhao J, Guo P, Wang D, Zhao D, Ren T, et al (2014). Clinical analyses of primary primitive neuroectodermal tumors in the female genital tract. Int J Gynecol Cncer.

[25] Jain S, Xu R, Prieto VG, Lee P (2010;23). Molecular classification of soft tissue sarcomas and its clinical applications. Int J Cain Exp Pathol.

[26] Llombart-Bosch A, Machado I, Navarro S, Bertoni F, Bacchini P, Alberghini M, et al (2009). Histological heterogeneity of Ewing’s sarcoma/PNET: an immunohistochemical analysis of 415 genetically confirmed cases with clinical support. Virchows Arch.

[27] Young RH, Oliva E, Scully RE (1994). Small cell carcinoma of the ovary, hypercalcemic type. A clinicopathological analysis of 150 cases. Am J Surg Pathol.

[28] Folpe AL, Hill CE, Parham DM, O’Shea PA, Weiss SW (2000). Immunohistochemical detection of FLI-1 protein expression: a study of 132 round cell tumors with emphasis on CD99-positive mimics of Ewing’s sarcoma/primitive neuroectodermal tumor. Am J Surg Pathol.

[29] Ferguson SE, Gerald W, Barakat RR, Chi DS, Soslow RA (2007). Clinicopathologic features of rhabdomyosarcoma of gynecologic origin in adults. Am J Surg Pathol.

